# Supplementary material for: Small Cationic Cysteine-Rich Defensin-Derived Antifungal Peptide Controls White Mold in Soybean
Source: J Fungi (Basel). 2023 Aug 24;9(9):873. doi: 10.3390/jof9090873 (PMC10532163; doi:10.3390/jof9090873)
Supplement: Supplementary file 1 [file jof-09-00873-s001.zip › jof-2428210-supplementary.pdf]

Supporting Information

Table S1. Amino acid sequences of GMA4CG_V6 and its variants.

Peptide	Sequence	Disulfide bond
GMA4CG_V6	GGRCKGFRRRWFWTRIC	C14-C17
GMA4CG_V6_lin	GGRCKGFRRRWFWTRIC	None
GMA4CG_V6_Ala 3	GGRCKGFAAAAFWTRIC	None

Table S2. *S. sclerotiorum* gene-specific primers used for qPCR analysis of gene expression.

Gene	Forward primer (5′-3′)	Reverse primer (5′-3′)	Reference for primers
cna1	GATGCTGGTTACCGAATG	AGCGGCTTTGTTGTTGTA	Wang et al., 2019
Pac1	TGCTGGTTCACCTTCTGTT	CAGGCTTCTCCTCAGTCTTTA	
Pka2	GGCATTTACAGAAAGAGGG	ATAATCCAAAGTCGCACAA	
Smk1	ATGTATGGTCTGTTGGGTGTA	GATTTGATGCCGTAGTAATCT	
actin	CCCCAGCGTTCTACGTCT	CATGTCAACACGAGCAATG	
β-tubu-lin	TTGGATTTGCTCCTTTGACCAG	AGCGGCCATCATGTTCTTAGG	Harel et al.,2006

Table S3. EC_{50} and MIC values of GMA4CG_V6 for *S. sclerotiorum 555.*

Fungi	EC_{50} (μM)	MIC (μM)
S. sclerotiorum 555	14	24
S. sclerotiorum 1902	14	24
S. sclerotiorum 1922	14	24

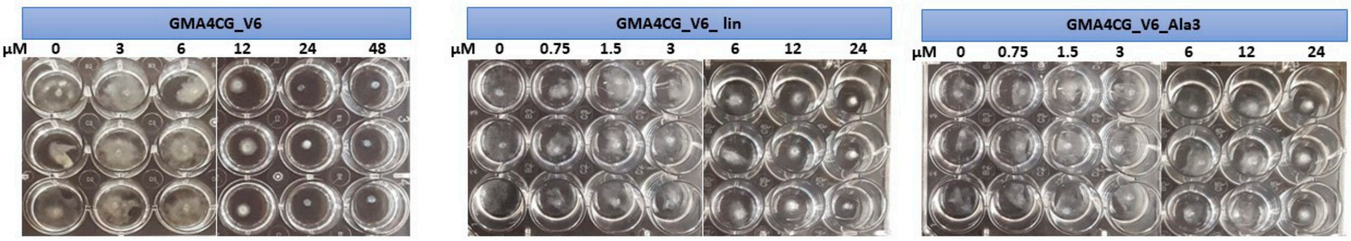

Figure S1. *In vitro* antifungal activity of GMA4CG_V6 variants against *S. sclerotiorum 555*. Representative pictures showing the antifungal activity of GMA4CG_V6, GMA4CG_V6_lin and GMA4CG_V6_Al3 against *S. sclerotiorum 555, 1902* and *1922* in SFM media.

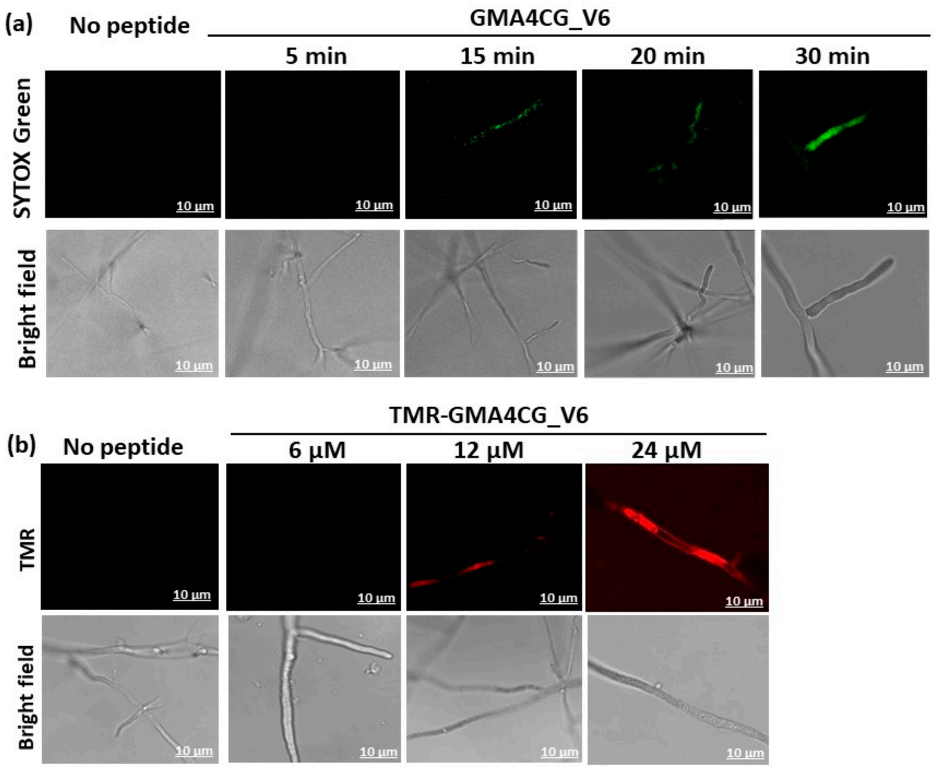

Figure S2. Membrane permeabilization activity and uptake of sub-lethal concentrations of GMA4CG_V6 by fungal cells. **(a)** Confocal microscopy images and corresponding bright field images of SG uptake in *S. sclerotiorum 555* hyphae treated with 24 μM GMA4CG_V6 within 30 min. (Scale bar, 10 μM). **(b)** The intracellular localization of 6, 12 and 24 μM TMR-labeled GMA4CG_V6 in *S. sclerotiorum 555*. The confocal microscope images were captured 2-5 min after TMR-GMA4CG_V6 challenge.
